# Prevalence and predictors of advance directive among terminally ill patients in Taiwan before enactment of Patient Right to Autonomy Act: a nationwide population-based study

**DOI:** 10.1186/s12904-022-01069-1

**Published:** 2022-10-12

**Authors:** Hui Yu Chang, Naomi Takemura, Pui Hing Chau, Chia-Chin Lin

**Affiliations:** 1 School of Nursing, College of Nursing, Taipei Medical University, New Taipei City, Taiwan; 2 Cancer Center, Far Eastern Memorial Hospital, New Taipei City, Taiwan; 3 School of Nursing, Li Ka Shing Faculty of Medicine, The University of Hong Kong, 5/F, 3 Sassoon Road, Hong Kong, Pokfulam China; 4 Alice Ho Miu Ling Nethersole Charity Foundation Professor in Nursing, Hong Kong, China

**Keywords:** Advance directives, Advance care planning, End-of-life, Terminally ill

## Abstract

**Background:**

Signing advance directives (ADs) ensures that terminally ill patients receive end-of-life care, according to their wishes, thereby promoting human dignity and sparing them from unnecessary suffering. Despite the enactment of the Hospice Palliative Care Act in Taiwan in 2000, the completion rates of ADs have been found to be low among patients with chronic illness conditions. To date, limited existing research is available regarding the factors associated with AD completion in terminally ill patients in Taiwan. To explore signed AD characteristics, compare differences in signing ADs between patients with and without cancer, and examine the factors associated with signing ADs in terminally ill patients.

**Methods:**

A nationwide study was conducted using data collected via a retrospective review of medical death records from 18 randomly selected hospitals in the northern, central, and southern parts of Taiwan. We collected 200 records, including both cancer and non-cancer-related deaths, from each hospital. Univariate and multivariate logistics regressions were conducted to examine factors associated with signing advance directives among all patients- with and without cancer.

**Results:**

Among the 3004 reviewed medical records, 79% had signed ADs, with most (95%) being signed by patients’ caregivers. A higher education level (OR = 1.52, 95% CI = 1.10, 2.08, *p* = 0.010); cancer diagnosis (OR = 2.37, 95% CI = 1.79, 3.16, *p* < 0.001); having family members (OR = 5.62, 95% CI = 2.95, 10.69, *p* < 0.001), care homes (OR = 4.52, 95% CI = 1.97, 10.38, *p* < 0.001), friends, or maids (OR = 3.82, 95% CI = 1.76, 8.29, *p* = 0.001) as primary caregivers; and patients knowing about their poor prognosis (OR = 15.39, 95% CI = 5.66, 41.83, *p* < 0.001) were associated with a higher likelihood of signing ADs.

**Conclusions:**

Patients with non-malignant chronic illnesses were less likely to have ADs signed by either patients or family caregivers than those with cancer, with the lowest likelihood observed in patients with cardiovascular diseases. Whenever possible, primary caregivers should be involved in discussing ADs with patients, and the importance of truth telling should be reinforced. Following these principles, each patient’s end-of-life care preferences can be respected, thereby promoting quality of care before the patient’s death.

## Background

Advance directive (AD) is a legal document that encompasses living wills, durable power of attorney for health care, or a combination of both [1]. It proposes any future healthcare decisions that may be required when individuals lose their decision-making capacity. An AD ensures that critically or terminally ill patients receive end-of-life (EOL) care, as their wishes, and they can avoid unnecessary or aggressive EOL medical treatment [2]. Previous studies reported that written ADs were associated with less life-sustaining treatment in the last month of life, greater use of hospice care, and lower likelihood of terminal hospitalization [3, 4]. Written ADs were also positively associated with a tendency for improvements in perceived EOL care quality, such as fewer concerns regarding physician communication, better management of family expectations while dying, and enhanced quality of death [5]. Thus, this approach can ensure appropriate EOL care and peaceful death [6], thereby promoting human dignity and sparing patients unnecessary suffering.

In many countries, EOL care focuses mainly on patients with cancer as cancer patients usually experienced a short decline in their illness trajectories [7]. However, there is a need to extend EOL care to non-cancer patients, who are susceptible to multiple physical and psychological symptoms, similar to those observed in cancer patients [8]. Particularly, patients with deteriorating end-stage chronic illness suffer from increased dependence and social isolation, thereby enhancing their families’ burden [9]. Therefore, patients with terminal illness—cancer or end-stage chronic illness—should receive appropriate and high-quality EOL care. Healthcare professionals expressed difficulties in determining the optimal time to discuss EOL care with other terminally ill patients due to the unpredictable illness trajectory; as a result, EOL discussions with the patient tend to occur merely few days before death [10]. Furthermore, patients’ changing attitudes amidst the illness trajectory and the varying preferences for optimal timing impede the commencement of EOL discussion [11]. Although terminal cancer usually imposes a linear decline in health status, discrepancies do exist in the optimal time for AD implementation, as physicians consider terminal diagnosis, while patients and their caregivers prefer earlier times when symptoms are not noticeable [12].

Individual sociodemographic variables and health status have demonstrated an association with the acceptance and completion of AD in the general population. Prior studies reported AD completion to be associated with older age [13–15], female sex [13, 14], higher education [14, 16, 17], and higher income [16]. Moreover, those with poorer health status, particularly those with critical illness or chronic medication use reported associations with higher rates of AD completion [15, 18]. While most studies were conducted in non-Asian countries and among the general population, there is a need to examine factors associated with AD completion in terminally ill patients in Taiwan to account for cultural differences. The Hospice Palliative Care Act was enacted in Taiwan in 2000 providing patients and their family surrogates with the right to refuse life-sustaining treatment; this act declared Taiwan the first Asian region to facilitate natural death for those who desired it [19]. Although the act enshrined patients’ right to sign a “Do Not Resuscitate” (DNR) order and draft an AD, the completion rates of ADs in Taiwan have been found to be low among patients with chronic illness conditions [20, 21]. Under these circumstances, the rate of AD completion can be promoted by obtaining a thorough understanding of the factors associated with drafting ADs. In this study, we aimed to explore the characteristics of signed ADs, compare the differences in signing ADs between patients with and without cancer, and to examine the factors associated with signing ADs in terminally ill patients in Taiwan.

## Conceptual framework

Andersen’s health behavior model was used to guide this analysis [22]. This model was commonly used to investigate health service utilization and can offer insight into health-related human behavior to explain why people take certain course of action [22]. This study has adopted the three major components of Andersen’s behavior model: predisposing, enabling, and need factors. Predisposing factors contribute to a person’s tendency to choose and perform a specific behavior. Such factors relate to sociodemographic characteristics, such as age, sex, education, marital status, and religion. Enabling factors refer to resources that facilitate a behavior, as well as social relationships. Patients’ primary caregivers was included in this study as having social network allows patients to learn more about and share their views on ADs. Need factors pertain to the presence of illnesses or disabilities and perceived health service needs as people do not need to access to any health services when they are healthy. Therefore, patients’ awareness of their poor prognosis and the type of disease diagnosis were included in the analysis as need factors.

## Methods

### Study design and procedure

This is a population-based study, in which hospitals in the major administrative regions in Taiwan were included. A retrospective review of the death medical records from various hospitals was conducted. In total, 18 hospitals (either medical centers or metropolitan hospitals based on the National Hospital Accreditation System) were randomly selected from the northern, central, and southern parts of Taiwan. We obtained 200 records randomly from each hospital, half of whom were diagnosed with cancer according to the International Classification of Diseases-9 [23] and the other half were diagnosed with non-cancer death. Therefore, medical records retrieved were representative of the whole Taiwan population. The medical records were included in this study, if: 1) patients died during hospitalization from July 1st 2004 to October 30th 2009 and 2) deaths were not related to suicide or accidents. The data coding and review were completed by two trained and experienced researchers. Data extraction was performed based on the data extraction form developed by the team comprising the experts in the palliative care field. The data reviewer extracted the relevant items according to the form. Some codes were predetermined as in the data extraction form, while a new code was determined within the research team if the data was not listed in the predetermined codes. Confidentiality was ensured by adopting review methods based on the “Guidelines for Collection and Use of Human Specimens for Research” from the Ministry of Health and Welfare, Taiwan. Additionally, we “de-linked” the data, i.e., a process to completely and permanently destroy the identifiable code and corresponding data serving to identify the dead person. The medical records with missing information were excluded from the respective analyses. Ethics approval was obtained from the Institutional Review Board of the Taipei Medical University Hospital (TMUH-01-07-08).

### Study instruments

#### Retrospective review of medical death records

Eight experts, including nurses and physicians experienced in palliative care, designed and validated the instrument “The current state of signing advance directives in medical records” based on current evidence and expert advice. Each expert rated the relevance of each item on a 5-point Likert scale, with a higher score indicating a higher relevance. The item-level content validity index (CVI) was calculated as the proportion of 3 or above ratings by the experts, and an item-level CVI of 0.8 or above was considered acceptable [24]. The average of the item-level CVI reached 0.95. The instrument comprised two parts: (a) patient demographics and (b) the characteristics of signed AD. Patient demographics included sex, age, medical diagnosis, education level, marital status, religion, and primary caregiver. The characteristics of signed AD included questions inquiring patients’ awareness of their disease condition (yes, no, or not recorded), the reasons of signing an AD, the key person initiating an AD, the relationship between patient and the person signing, patients’ condition upon signing, whether specific life-sustaining treatments (endotracheal intubation, mechanical ventilation, resuscitative drugs, cardiopulmonary resuscitation, cardiac defibrillation, pacemaker, and other resuscitation procedures) were executed according to ADs, and whether letter of intent was withdrawn after signing.

### Statistical analysis

Participants’ sociodemographic and clinical characteristics and AD characteristics were presented using descriptive statistics. Patient demographic and clinical characteristics were compared using the exact chi-squared test between cancer and non-cancer patients, and two-independent sample t-tests between these patients and AD signers. Univariate and multivariate logistic regressions were conducted to examine factors associated with signing ADs among all patients—with and without cancer. Univariate regression analysis was performed to examine the association of each factor with signing ADs. Subsequently, the following factors (predisposing factors- age, sex, education, marital status, and religion; enabling factor-patients’ primary caregivers, and need factors- patients’ awareness of their poor prognosis and the type of disease diagnosis) were added in the multivariable regression models. The presence of multicollinearity was examined in these models. The results were presented using odds ratios (ORs) and 95% confidence intervals (CIs). Medical records with missing data were excluded from the corresponding analyses. All analyses were performed using the Statistical Package for Social Sciences (SPSS, v.25.0, IBM SPSS Statistics, IBM Corporation). Statistical significance was set at *P* < 0.05 (two-sided).

## Results

### Patients’ demographic characteristics in medical death records

Medical death records (*n* = 3004) were retrospectively reviewed and analyzed. Of the 3004 patients, 2369 (79%) had completed ADs. Table 1 shows patients’ demographic characteristics in the reviewed medical death records. Patients’ mean age was 66.26 (16.35) years. Most patients in the records were male (62%), with 0–6 years of education (56%), married (71%), and religious (79%), and were unaware of their condition or their results were not recorded (80%). Over half (59%) of the patients were diagnosed with cancer, with family members as the primary caregivers (86%). Most patients were diagnosed with liver cancer (21%), followed by lung cancer (18%), whereas most non-cancer patients were diagnosed with cerebrovascular diseases (21%), followed by respiratory diseases (20%). Patients demonstrated significant differences in age (*p* < 0.001), sex (*p* < 0.001), years of education (*p* < 0.001), marital status (*p* < 0.001), religion (*p* = 0.001), primary caregiver (*p* = 0.001), status of AD (*p* < 0.001), and patients’ awareness of their poor prognosis (*p* < 0.001). Table 2 presents the demographic characteristics of patients with signed ADs: among the 2369 AD signers, 1517 (64%) and 852 (36%) were patients with and without cancer, respectively. Demographic characteristics of patients, who signed ADs, were similar to those of all patients (Table 2), with significant differences in age (*p* < 0.001), sex (*p* = 0.001), years of education (*p* < 0.001), religion (*p* = 0.004), primary caregiver (*p* < 0.001), and patients’ awareness of their poor prognosis (*p* < 0.001) among patients with and without cancer.

**Table 1:** Demographic characteristics of medical death records

	Total (***n*** = 3004)	Cancer patients (***n*** = 1781)	Non-cancer patients (***n*** = 1223)	*p*-value^a^
	Mean (SD)	Mean (SD)	Mean (SD)	
Age, years	66.26 (16.35)	64.34 (14.87)	69.08 (17.93)	< 0.001*
	N (%)	N (%)	N (%)	*p*-value^a^
Gender				< 0.001*
Male	1858 (62)	1151 (65)	707 (58)	
Female	1143 (38)	630 (35)	513 (42)	
Years of education				< 0.001*
0–6	1674 (56)	899 (58)	775 (70)	
≥ 7	985 (33)	658 (42)	327 (30)	
Marital status				< 0.001*
Married	2119 (71)	1343 (75)	776 (64)	
Not married	754 (25)	360 (21)	394 (33)	
Religion				0.001*
No	637 (21)	337 (19)	300 (25)	
Yes	2371 (79)	1444 (81)	923 (75)	
Major caregiver				0.001*
Family members	2482 (86)	1531 (86)	951 (78)	
Maids	161 (6)	99 (6)	62 (5)	
Care homes^b^	128 (4)	32 (2)	96 (8)	
Friends	26 (1)	10 (1)	16 (1)	
Self^c^	79 (3)	39 (2)	40 (3)	
Status of AD				< 0.001*
With AD	2369 (79)	1517 (88)	852 (72)	
Without AD	537 (18)	202 (12)	335 (28)	
Patients know their poor prognosis				< 0.001*
Yes	559 (20)	469 (28)	90 (8)	
No/not recorded	2202 (80)	1212 (72)	990 (92)	
Type of cancer diagnosis				–
Brain/CNS Lymphoma	–	27 (2)	–	
Breast cancer	–	68 (4)	–	
Cervical cancer	–	43 (2)	–	
Cholangiocarcinoma	–	29 (2)	–	
Colorectal cancer	–	172 (10)	–	
Esophageal cancer	–	81 (5)	–	
Head and neck cancer		156 (9)		
Hematological malignancies	–	104 (6)	–	
Liver cancer	–	370 (21)	–	
Lung cancer	–	319 (18)	–	
Ovarian cancer	–	28 (2)	–	
Pancreatic cancer	–	63 (4)	–	
Prostate cancer	–	40 (2)	–	
Stomach cancer	–	110 (6)	–	
Urological cancer	–	62 (4)	–	
Other cancer	–	93 (5)	–	
Type of non-cancer diagnosis				–
Cardiovascular diseases	–	–	109 (9)	
Cerebrovascular diseases	–	–	252 (21)	
Chronic liver diseases	–	–	138 (11)	
Multiple organ failure	–	–	29 (2)	
Renal diseases	–	–	104 (9)	
Respiratory diseases	–	–	249 (20)	
Rheumatic diseases	–	–	21 (2)	
Sepsis/ septic shock	–	–	199 (16)	
Other chronic diseases	–	–	86 (7)	

*p < 0.05

^a^Obtained by two-independent samples t-test or by exact chi-square tests examining the difference in demographic characteristics between cancer and non-cancer patients

^b^Included 8 non-cancer patients who reported both care homes and family members

^c^Included 1 cancer patient who reported both self and family members

**Table 2:** Demographic characteristics of medical death records with signed advance directives

	Total (***n*** = 2369)	Cancer patients (***n*** = 1517)	Non-cancer patients (***n*** = 852)	***p***-value^**a**^
	Mean (SD)	Mean (SD)	Mean (SD)	
Age, years	66.43 (16.17)	64.62 (14.81)	69.67 (17.91)	< 0.001*
	N (%)	N (%)	N (%)	*p*-value^a^
Gender				0.001*
Male	1460 (62)	975 (64)	485 (57)	
Female	907 (38)	542 (36)	365 (43)	
Years of education				< 0.001*
0–6	1343 (57)	787 (58)	556 (70)	
≥ 7	839 (35)	598 (42)	241 (30)	
Marital status				0.119
Married	2101 (90)	1358 (90)	743 (87)	
Not married	245 (10)	146 (10)	99 (12)	
Religion				0.004*
No	638 (27)	294 (19)	208 (24)	
Yes	1713 (72)	1223 (81)	644 (76)	
Major caregiver				< 0.001*
Family members	1979 (94)	1300 (86)	679 (80)	
Maids	134 (6)	93 (6)	41 (5)	
Care homes^b^	94 (4)	26 (2)	66 (8)	
Friends	11 (1)	9 (1)	2 (0.2)	
Self^c^	36 (2)	26 (2)	10 (1)	
Patients know their poor prognosis				< 0.001*
Yes	535 (23)	448 (30)	87 (10)	
No/not recorded	1834 (77)	1069 (71)	765 (90)	
Type of cancer diagnosis				–
Brain/CNS Lymphoma	–	22 (2)	–	
Breast cancer	–	60 (4)	–	
Cervical cancer	–	38 (3)	–	
Cholangiocarcinoma	–	27 (2)	–	
Colorectal cancer	–	152 (10)	–	
Esophageal cancer	–	66 (4)	–	
Head and neck cancer		135 (9)		
Hematological malignancies	–	88 (6)	–	
Liver cancer	–	309 (20)	–	
Lung cancer	–	271 (18)	–	
Ovarian cancer	–	27 (2)	–	
Pancreatic cancer	–	54 (4)	–	
Prostate cancer	–	36 (2)	–	
Stomach cancer	–	98 (7)	–	
Urological cancer	–	56 (4)	–	
Other cancer	–	78 (5)	–	
Type of non-cancer diagnosis				–
Cardiovascular diseases	–	–	57 (7)	
Cerebrovascular diseases	–	–	197 (23)	
Chronic liver diseases	–	–	104 (12)	
Multiple organ failure	–	–	21 (3)	
Renal diseases	–	–	69 (8)	
Respiratory diseases	–	–	181 (21)	
Rheumatic diseases	–	–	14 (2)	
Sepsis/ septic shock	–	–	144 (17)	
Other chronic diseases	–	–	65 (8)	

*p < 0.05

^a^Obtained by two-independent samples t-test or by exact chi-square tests examining the difference in demographic characteristics between cancer and non-cancer patients

^b^Included 6 non-cancer patients who reported both care homes and family members

^c^Included 1 cancer patient who reported both self and family members

### Characteristics of signed advance directives in medical death records

Table 3 summarizes the characteristics of the signed ADs, for patients with and without cancer: on average, patients signed ADs, 8.45 (19.97) days before death. Most directives were signed by patients’ caregivers (95%), who were primarily patients’ children (60%) with the major reasons of signing being poor prognosis (52%) and imminent death (46%). Moreover, 35 and 34% of the patients were in a comatose and conscious state, respectively, when ADs were signed. Doctors were the key persons initiating ADs (71%), and most ADs were complied with (99%). Specifically, the majority of life-sustaining treatments, such as endotracheal intubation (91%), mechanical ventilation (77%), resuscitative drugs (69%), cardiopulmonary resuscitation (97%), cardiac defibrillation (93%), pacemaker (81%), and other resuscitation procedures (62%) were performed or not performed according to ADs. There were significant differences between patients with and without cancer in the following scenarios: a) when ADs were signed due to poor prognosis, imminent death, patient’s intentions, incurable pain (*p* < 0.001), and when this was initiated by family members (*p* = 0.046), or to avoid patients’ suffering (*p* = 0.040); b) when the palliative care team and patients themselves were the key persons initiating an AD (*p* < 0.001); c) in the relationship between patients and people signing an AD(*p* < 0.001); d) and patients’ consciousness level when signing the AD (*p* < 0.001).

**Table 3:** Characteristics of signed advance directives

	Total (***n*** = 2369)	Cancer patients (***n*** = 1517)	Non-cancer patients (***n*** = 852)	***p***-value^**a**^
	Mean (SD)	Mean (SD)	Mean (SD)	
Number of days till death after AD signed	8.45 (19.97)	8.61 (16.11)	8.18 (25.36)	0.616
	N (%)	N (%)	N (%)	*p*-value^a^
**Reason of signing an AD^**				
Poor prognosis	1234 (52)	840 (55)	394 (46)	< 0.001*
Close to death	1095 (46)	604 (40)	491 (58)	< 0.001*
Initiated by family members	349 (15)	207 (14)	142 (17)	0.046*
Do not want patients to suffer	183 (8)	130 (9)	53 (6)	0.040*
Patient’s intention	157 (7)	140 (9)	17 (2)	< 0.001*
Incurable pain	102 (4)	94 (6)	8 (1)	< 0.001*
Old age	75 (3)	42 (3)	33 (4)	0.141
Financial difficulties	10 (0.4)	6 (0.4)	4 (0.5)	0.790
Others	4 (0.2)	1 (0.1)	3 (0.4)	0.103
**Key person initiating an AD^**				
Doctors	1679 (71)	1059 (70)	620 (73)	0.128
Family members	303 (13)	180 (12)	123 (14)	0.072
Palliative care teams	164 (7)	163 (11)	1 (0.1)	< 0.001*
Nurses	102 (4)	72 (5)	30 (4)	0.159
Self	50 (2)	44 (3)	6 (1)	< 0.001*
Social workers	3 (0.1)	3 (0.2)	0 (0)	0.194
Not recorded	289 (12)	181 (12)	108 (13)	0.595
**Relationship between patients and person signing an AD**				< 0.001*
Children	1429 (60)	876 (58)	553 (65)	
Spouses	482 (20)	351 (23)	131 (15)	
Grandchildren	26 (14)	13 (0.8)	19 (2)	
Siblings	126 (5)	72 (5)	54 (6)	
Parents	84 (4)	43 (3)	41 (5)	
Self	95 (4)	92 (6)	3 (0.4)	
Daughters in-law	47 (2)	24 (2)	23 (3)	
Other relatives	29 (1)	16 (1)	13 (1)	
Not recorded	31 (1)	21 (1)	10 (1)	
**Patients’ conscious level when signing**				< 0.001*
Coma	829 (35)	300 (20)	529 (62)	
Conscious	812 (34)	715 (47)	97 (11)	
Dozing	269 (11)	213 (14)	56 (7)	
Half-coma	171 (7)	90 (6)	81 (10)	
Confusion	159 (7)	115 (8)	44 (5)	
Dementia	10 (0.4)	4 (0.3)	6 (0.7)	
Not recorded	75 (3)	59 (4)	16 (2)	
**Durable power of attorney**				0.567
Yes	70 (3)	46 (3)	24 3)	
No	2299 (97)	1471 (97)	828 (97)	
**Compliance to ADs**^**b**^				0.563
Yes^c^	2355 (99)	1507 (99)	848 (99)	
No^d^	14 (1)	10 (1)	4 (1)	
**Specific end-of-life care executed according to ADs ^**				
***Endotracheal intubation***				< 0.001*
Executed according to AD	19 (1)	11 (1)	8 (3)	
Not executed according to AD	1355 (90)	1110 (93)	245 (81)	
Executed not according to AD	8 (1)	7 (1)	1 (0.3)	
Not executed not according to AD	120 (8)	72 (6)	48 (16)	
***Mechanical ventilation***				< 0.001*
Executed according to AD	46 (3)	34 (3)	12 (4)	
Not executed according to AD	1100 (74)	919 (78)	181 (61)	
Executed not according to AD	59 (4)	38 (3)	21 (7)	
Not executed not according to AD	274 (19)	192 (16)	82 (28)	
***Resuscitative drugs***^***e***^				< 0.001*
Executed according to AD	582 (25)	288 (19)	294 (35)	
Not executed according to AD	1034 (44)	772 (51)	262 (31)	
Executed not according to AD	160 (7)	96 6)	64 (8)	
Not executed not according to AD	591 (25)	359 (24)	232 (27)	
***Cardiopulmonary resuscitation***				
Executed according to AD	15 (1)	4 (0.3)	11 (1)	0.010*
Not executed according to AD	2269 (96)	1457 (96)	812 (95)	
Executed not according to AD	26 (1)	20 (1)	6 (1)	
Not executed not according to AD	57 (2)	34 (2)	23 (3)	
***Cardiac defibrillation***				0.030*
Executed according to AD	2 (0.1)	1 (0.1)	1 (0.1)	
Not executed according to AD	2195 (93)	1423 (94)	772 (91)	
Executed not according to AD	7 (0.3)	4 (0.3)	3 (0.4)	
Not executed not according to AD	163 (7)	87 (6)	76 (9)	
***Pacemaker***				N/A
Executed according to AD	2 (0.1)	0 (0)	2 (0.2)	
Not executed according to AD	1928 (81)	1286 (85)	642 (75)	
Executed not according to AD	1 (0.04)	1 (0.1)	0 (0)	
Not executed not according to AD	435 (18)	228 (15)	207 (24)	
***Other resuscitation procedures***				N/A
Executed according to AD	5 (0.2)	1 (0.1)	4 (0.5)	
Not executed according to AD	1475 (62)	982 (65)	493 (58)	
Executed not according to AD	3 (0.1)	3 (0.2)	0 (0)	
Not executed not according to AD	884 (37)	529 (35)	355 (42)	

^ The number of patients having the specific care in their ADs varied

*Abbreviations*: *AD* Advance directives, *SD* Standard deviation

*p < 0.05

^a^Obtained by two-independent samples t test or by exact chi-square tests examining the difference in characteristics of signed advance directives between cancer and non-cancer patients

^b^At least one of the EOL care procedures were executed or not executed according to AD

^c^Executed or not executed according to AD

^d^Executed or not executed not according to AD

^e^Resuscitative drug means aminophylline, epinephrine, sodium bicarbonate, nitroglycerin, diphenhydramine, hydrocortisone, amiodarone

### Factors associated with signing advance directives among patients with and without cancer

Table 4 shows the univariate and multivariate regression models for all the patients. Accordingly, ADs were more likely to signed by patients who were married (OR = 1.88, 95% CI = 1.45, 2.43, *p* < 0.001), had more years of education (OR = 1.42, 95% CI = 1.15, 1.75, *p* = 0.001); were religious (OR = 1.25, 95% CI = 1.00, 1.55, *p* = 0.05); were diagnosed with cancer (OR = 2.95, 95% CI = 2.43, 3.58, *p* < 0.001); and had family members (OR = 5.34, 95% CI = 3.34, 8.53, *p* < 0.001), care homes (OR = 3.66, 95% CI = 1.97, 6.82, *p* < 0.001), friends, or maids (OR = 4.72, 95% CI = 2.62, 8.51, *p* < 0.001) as primary caregivers; and who knew about their poor prognosis (OR = 17.79, 95% CI = 7.32, 43.29, *p* < 0.001). After adjusting for factors, more years of education (OR = 1.52, 95% CI = 1.10, 2.08, *p* = 0.010), and cancer diagnosis (OR = 2.37, 95% CI = 1.79, 3.16, *p* < 0.001) were associated with a higher likelihood of having ADs signed. Compared caring for oneself, having family members (OR = 5.62, 95% CI = 2.95, 10.69, *p* < 0.001), care homes (OR = 4.52, 95% CI = 1.97, 10.38, *p* < 0.001), friends, or maids (OR = 3.82, 95% CI = 1.76, 8.29, *p* = 0.001) as primary caregivers were associated with a higher likelihood of having ADs signed. Additionally, the likelihood of having ADs signed increased when patients were aware of their poor prognosis (OR = 15.39, 95% CI = 5.66, 41.83, *p* < 0.001).

**Table 4:** Logistic regression of signing advance directives among all patients

	Crude odds ratio (95% CI)	***p***-value	Adjusted odds ratio (95% CI)	***p***-value
**Age**	1.00 (0.99, 1.01)	0.939	1.01 (1.00, 1.02)	0.170
**Gender**				
Male (vs female)	0.89 (0.73, 1.08)	0.247	0.96 (0.72, 1.27)	0.761
**Marital status**				
Married (vs not married)	1.88 (1.45, 2.43)	< 0.001*	1.13 (0.71, 1.78)	0.612
**Years of education**				
≥ 7 (vs 0–6)	1.42 (1.15, 1.75)	0.001*	1.52 (1.10, 2.08)	0.010*
**Religion**				
Yes (vs no)	1.25 (1.00, 1.55)	0.046*	1.23 (0.90, 1.66)	0.190
**Diagnosis**				
Cancer (vs non-cancer)	2.95 (2.43, 3.58)	< 0.001*	2.37 (1.79, 3.16)	< 0.001*
**Major caregiver**				
Family members (vs self)	5.34 (3.34, 8.53)	< 0.001*	5.62 (2.95, 10.69)	< 0.001*
Care homes (vs self)	3.66 (1.97, 6.82)	< 0.001*	4.52 (1.97, 10.38)	< 0.001*
Friends/maid (vs self)	4.72 (2.62, 8.51)	< 0.001*	3.82 (1.76, 8.29)	0.001*
**Patients know their poor prognosis**				
Yes (vs no)	17.79 (7.32, 43.29)	< 0.001*	15.39 (5.66, 41.83)	< 0.001*

*p < 0.05

*Abbreviations*: *CI* Confidence interval

Tables 5 and 6 present the univariate and multivariate regression models for patients with and without cancer, respectively. Among patients with cancer, male patients (OR = 0.73, 95% CI = 0.53, 1.00, *p* = 0.05) were less likely to have ADs signed (Table 5). Patients with more years of education (OR = 1.42, 95% CI = 1.02, 1.98, *p* = 0.039); having family members (OR = 2.99, 95% CI = 1.41, 6.33, *p* = 0.004), friends, or maids (OR = 8.08, 95% CI = 2.54, 25.76, *p* < 0.001) as primary caregivers rather than themselves; and aware of their poor prognosis (OR = 10.90, 95% CI = 3.99, 29.76, *p* < 0.001) were more likely to have ADs signed. After adjusting for factors, having family members (OR = 3.48, 95% CI = 1.30, 9.33, *p* = 0.013), friends, or maids (OR = 9.21, 95% CI = 1.69, 50.09, *p* = 0.010) as primary caregivers rather than caring for themselves; and patients’ being aware of their poor prognosis (OR = 11.85, 95% CI = 4.27, 32.89, *p* < 0.001) were associated with a higher likelihood of having ADs signed.

**Table 5:** Logistic regression of signing advance directives among cancer patients

	Cancer patients			
	Crude odds ratio (95% CI)	***p***-value	Adjusted odds ratio (95% CI)	***p***-value
**Age**	1.00 (0.99, 1.01)	0.466	1.01 (0.99, 1.03)	0.242
**Gender**				
Male (vs female)	0.73 (0.53, 1.00)	0.05*	0.73 (0.43, 1.26)	0.257
**Marital status**				
Married (vs not married)	1.44 (0.93, 2.24)	0.102	1.36 (0.67, 2.75)	0.390
**Years of education**				
≥ 7 (vs 0–6)	1.42 (1.02, 1.98)	0.039*	1.62 (1.00, 2.61)	0.051
**Religion**				
Yes (vs no)	1.13 (0.79, 1.61)	0.522	1.36 (0.83, 2.23)	0.221
**Major caregiver**				
Family members (vs self)	2.99 (1.41, 6.33)	0.004*	3.48 (1.30, 9.33)	0.013*
Care homes (vs self)	1.73 (0.55, 5.48)	0.349	1.29 (0.32, 5.15)	0.721
Friends/maid (vs self)	8.08 (2.54, 25.76)	< 0.001*	9.21 (1.69, 50.09)	0.010*
**Patients know their poor prognosis**				
Yes (vs no)	10.90 (3.99, 29.76)	< 0.001*	11.85 (4.27, 32.89)	< 0.001*
**Type of cancer diagnosis**				
Brain/CNS Lymphoma	0.55 (0.20, 1.55)	0.260	0.81 (0.16, 4.11)	0.801
Breast cancer	1.26 (0.50, 3.12)	0.626	0.79 (0.20, 3.17)	0.740
Cervical cancer	0.95 (0.35, 2.59)	0.926	0.31 (0.09, 1.11)	0.071
Cholangiocarcinoma	1.69 (0.39, 7.44)	0.485	1.44 (0.18, 11.74)	0.736
Colorectal cancer	1.19 (0.64, 2.23)	0.583	1.02 (0.39, 2.66)	0.966
Esophageal cancer	0.55 (0.28, 1.07)	0.080	0.66 (0.23, 1.87)	0.430
Head and neck cancer	0.81 (0.45, 1.44)	0.469	0.54 (0.22, 1.30)	0.168
Hematological malignancies	0.69 (0.36, 1.31)	0.257	0.58 (0.23, 1.48)	0.256
Liver cancer	1.11 (0.67, 1.83)	0.688	0.98 (0.46, 2.06)	0.951
Ovarian cancer	3.39 (0.45, 25.73)	0.238	0.73 (0.09, 6.27)	0.776
Pancreatic cancer	0.75 (0.34, 1.66)	0.481	0.79 (0.21, 2.99)	0.733
Prostate cancer	1.13 (0.38, 3.37)	0.828	1.23 (0.15, 10.09)	0.848
Stomach cancer	1.03 (0.51, 2.06)	0.946	1.05 (0.36, 3.08)	0.924
Urological cancer	1.17 (0.47, 2.92)	0.735	0.62 (0.19, 2.02)	0.427
Other cancer (vs lung cancer)	0.65 (0.34, 1.26)	0.203	0.77 (0.26, 2.29)	0.639

*p < 0.05

*Abbreviations*: *CI* Confidence interval, *CNS* Central nervous system

**Table 6:** Logistic regression of signing advance directives among non-cancer patients

	Non-cancer patients			
	Crude odds ratio (95% CI)	***p***-value	Adjusted odds ratio (95% CI)	***p***-value
**Age**	1.01 (1.00, 1.01)	0.174	1.01 (1.00, 1.02)	0.014*
**Gender**				
Male (vs female)	0.88 (0.68, 1.14)	0.328	0.93 (0.68, 1.25)	0.619
**Marital status**				
Married (vs not married)	1.95 (1.39, 2.74)	< 0.001*	0.99 (0.60, 1.62)	0.987
**Years of education**				
≥ 7 (vs 0–6)	1.10 (0.83, 1.48)	0.507	1.31 (0.93, 1.85)	0.119
**Religion**				
Yes (vs no)	1.17 (0.88, 1.56)	0.277	0.89 (0.64, 1.24)	0.488
**Major caregiver**				
Family members (vs self)	8.24 (3.96, 17.16)	< 0.001*	9.68 (3.99, 23.53)	< 0.001*
Care homes (vs self)	8.70 (3.66, 20.68)	< 0.001*	12.14 (4.29, 34.37)	< 0.001*
Friends/maid (vs self)	4.30 (1.82, 10.16)	0.001*	4.83 (1.81, 12.89)	0.002*
**Patients know their poor prognosis**				
Yes (vs no)	22.86 (3.17, 165.12)	0.002*	–	–
**Type of non-cancer diagnosis**				
Cerebrovascular diseases	3.27 (2.02, 5.28)	< 0.001*	3.90 (2.28, 6.66)	< 0.001*
Chronic liver diseases	2.79 (1.63, 4.79)	< 0.001*	3.13 (1.72, 5.68)	< 0.001*
Multiple organ failure	2.40 (0.98, 5.87)	0.056	2.60 (0.96, 7.07)	0.062
Renal diseases	1.80 (1.03, 3.13)	0.038*	2.11 (1.15, 3.87)	0.016*
Respiratory diseases	2.43 (1.52, 3.88)	< 0.001*	2.42 (1.45, 4.03)	0.001*
Rheumatic diseases	1.83 (0.68, 4.87)	0.230	2.60 (0.86, 7.81)	0.090
Sepsis/ septic shock	2.39 (1.47, 3.89)	< 0.001*	2.82 (1.63, 4.88)	< 0.001*
Other diseases (vs cardiovascular diseases)	2.82 (1.52, 5.24)	0.001*	3.08 (1.55, 6.13)	0.001*

*p < 0.05

*Abbreviations*: *CI* Confidence interval

Footnote: The variable “Patients know their poor prognosis” was not included in the analysis of non-cancer patients because there were too few cases in one level

As shown in Table 6, among non-cancer patients, those who were married (OR = 1.95, 95% CI = 1.39, 2.74, *p* < 0.001); had family members (OR = 8.24, 95% CI = 3.96, 17.16, *p* < 0.001), care homes (OR = 8.70, 95% CI = 3.66, 20.68, *p* < 0.001), friends, or maids (OR = 4.30, 95% CI = 1.82, 10.16, *p* = 0.001) as primary caregivers; and knew of their poor prognosis (OR = 22.86, 95% CI = 3.17, 165.12, *p* = 0.002) were more likely to have ADs signed. Patients with cerebrovascular diseases (OR = 3.27, 95% CI = 2.02, 5.28, *p* < 0.001), chronic liver disease (OR = 2.79, 95% CI = 1.63, 4.79, *p* < 0.001), renal diseases (OR = 1.80, 95% CI = 1.03, 3.13, *p* = 0.038), respiratory diseases (OR = 2.43, 95% CI = 1.52, 3.88, *p* < 0.001), sepsis or septic shock (OR = 2.39, 95% CI = 1.47, 3.89, *p* < 0.001), and other non-cancer diseases (OR = 2.82, 95% CI = 1.52, 5.24, *p* = 0.001) were more likely to have ADs signed than patients with cardiovascular diseases. After adjusting for factors, older age (OR = 1.01, 95% CI = 1.00, 1.02, *p* = 0.014); having family members (OR = 9.68, 95% CI = 3.99, 23.53, *p* < 0.001), care homes (OR = 12.14, 95% CI = 4.29, 34.37, *p* < 0.001), friends, or maids (OR = 4.83, 95% CI = 1.81, 12.89, *p* = 0.002) as primary caregivers rather than caring for themselves were associated with higher likelihood of having ADs signed. Additionally, patients with non-cancer diagnosis such as cerebrovascular diseases (OR = 3.90, 95% CI = 2.28, 6.66, *p* < 0.001), chronic liver diseases (OR = 3.13, 95% CI = 1.72, 5.68, *p* < 0.001), and multiple non-cancer diseases were associated with a higher likelihood of signing ADs than those with cardiovascular diseases.

## Discussion

To our knowledge, this is the first nationwide population-based study to examine the characteristics of signed ADs in the medical death records of Taiwan. Among the reviewed medical records, most patients (79%) had ADs signed by either patients or family caregivers, and the most frequent reasons for signing were the patients’ poor prognosis and their imminent death. The proportion of signed ADs in this study was higher than that in previous studies reporting 8.1–40.2% of signed ADs among Taiwanese older adults with chronic illness [15, 20, 25, 26]. A specific trigger was revealed for initiating EOL care preference or AD completion discussions: diagnosis of long-term or life-limiting conditions, such as cancer, or conditions with predicted loss of capacity [27–29]. Yang et al. indicated that the average time for executing ADs was when patients’ conditions became more critical or death was imminent [30]. In our study, all patients were either diagnosed with cancer or chronic diseases, and most ADs were signed when patients were in a comatose state or close to their death. These could plausibly explain the increased chances for patients or their families to consider EOL care-related issues, resulting in a higher prevalence of signed ADs. Another plausible reason would be the fact that our study only included those patients who deceased during the study period. In addition, this highlights the concern of ADs being signed at a late stage and might not facilitate the attainment of goal-concordant care. By acknowledging this, future EOL care intervention or practice can shift the focus from direct documentation to initiating conversation, that is, advance care planning. It is a process of communication that allows an individual to express their preferences for future medical care, without imposing the immediate need to sign any documents.

Interestingly, patients with cancer were more likely to have ADs signed by either patients or family caregivers than those without cancer. This finding is consistent with a systematic review suggesting that those with cancer diagnoses achieved higher rates of advance care planning than those with non-malignant life-limiting diagnoses [31]. This may be attributed to physicians’ perceptions of the increased reliability in predicting deaths of patients with cancer than those with non-malignant conditions [32]. Addition, terminal cancer patients typically demonstrate a linear decline in health status, resulting in the acceptance of EOL discussions and their imminent death. However, it is difficult to predict the illness trajectory for non-malignant chronic diseases, such as chronic obstructive pulmonary diseases, myocardial infarction, or stroke, and discrepancies exist in the preferences for optimal timing for EOL discussion [11]. Notably, patients diagnosed with cardiovascular diseases were less likely to sign ADs than most patients with other chronic diseases, supporting previous studies that reported that the rates of AD among patients with cardiac and pulmonary diseases were very low [33], even lower than those with other chronic illnesses [34]. Doctors were the key persons initiating an AD, and existing literature suggests cardiologists refer their patients for palliative care at much lower rates than other specialty professionals or they often refer patients at the terminal stage of their disease [35]. One reason for this is the difficulty in determining the prognosis of the illness trajectory in patients with heart failure, making it difficult to decide when and how palliative care will be most beneficial to patients [35]. A recently published Delphi study listed a wide range of criteria for specialist palliative care referral for patients with advanced heart failure [36]. Yet, the author stated it was just an initial step towards standardizing clinical care and future research is required for validation and implementation in cardiology care settings [36]. Another plausible reason was the hesitancy of physicians of patients with heart failure to participate in advance care planning discussions due to their concerns about taking away the hope from their patients and hastening their death [37]. Further research is warranted to explore the EOL care preferences and practices, particularly among patients with non-malignant chronic illness, as well as patients at different stages of their illness trajectory.

Among all patients, we observed an increased likelihood of ADs being signed when patients had more years of education and had family members, care homes, and friends, or maids as primary caregivers. Older age was also associated with a higher likelihood of ADs being signed in non-cancer patients. Moreover, consistent with previous studies our findings revealed an association between higher education and higher rates of AD completion [14, 16, 17]. Education is related to health literacy, which may further be correlated with higher economic status and increased awareness of advance care planning. This may result in the beneficial opportunity for more educated individuals to complete an AD. Caregivers are defined as individuals who play a substantial role in caring for and assisting in activities of daily living of patients [38]. Patients with life-limiting illness expressed their preferences for involving their caregivers in making EOL care decisions [39]; thus, caregivers’ participation is crucial in EOL care and decision-making [40]. Our findings supported that when family members, care homes, friends, or maids were the primary caregivers rather than patients themselves, patients were more likely to sign the ADs. Older adults living alone reported lower levels of AD perceptions than community-dwelling seniors [41], possibly due to social isolation, and the lack of communication emerging from unavoidable isolation as a result of their children’s independence and death of their spouses [41]. Older patients are commonly susceptible to more chronic comorbidities and are therefore more likely to have EOL care preference discussions and complete ADs [42]. Healthcare professionals, in particular the palliative care teams, should emphasize educating younger patients who live alone and those with non-malignant chronic illness in the importance of expressing their EOL care preferences and completing ADs when they are still conscious or in a less critical condition.

Disclosure of the patient’s condition is a crucial factor to be considered. In Taiwan, disclosing patients’ condition is a common medical dilemma for caregivers in hospice settings [43] as some believe that confidentiality regarding patients’ condition is a way of relieving patients’ burden. Meanwhile, studies have reported that patients who are aware of their condition are more likely to participate in healthcare decision-making and sign an AD [16, 30]. These results are consistent with our findings reporting that patients with poor prognosis, particularly those with cancer, were more likely to sign an AD. However, our findings may be require further investigation to examine the importance of truth telling in signing an AD and verify their accuracy.

Our results also demonstrated that most ADs in the reviewed records were signed by patients’ surrogates, who were mainly their children and spouses. Despite their conscious state, only 12% of the ADs were signed by the patients themselves, which is similar to prior studies demonstrating that approximately 91–98% of the EOL care-related decisions were made by family members [26, 44]. Additionally, this finding can be attributed to the predominance of family, as the primary unit of decision-making, in oriental cultures [45, 46]. Thus, EOL care decisions will not be made outside this fundamental social unit. Future clinical practice can include family members in discussing ADs, so as to respect the patient’s autonomy and refrain from overriding the patient’s opinion.

Furthermore, the Patient Right to Autonomy Act was passed by the Taiwanese legislature in 2015 and it was the first law in Asia to protect a patient’s right to autonomy including exercising the right to refuse medical treatments through ADs [47]. Several population-based studies revealed that the implementation of palliative care policies, namely the Patient Right to Autonomy Act in Taiwan was associated with improved palliative care utilization in regards to cancer and non-cancer diseases such as chronic obstructive pulmonary disease, dementia, and stroke [48–50]. This underscores the benefits of having ADs and therefore the importance of exploring the influential predictors of signing ADs in terminally ill patients.

### Implication for future research and practice

Implementation of ADs is vital for patients with cancer and those with other chronic illness and the general population to promote their autonomy and self-determination. Healthcare professionals should be better informed of hospitalized patients’ condition, who live alone with terminal illness, by introducing the idea of advance care planning and ADs at an earlier stage. This enables sufficient time for patients and/or their families to understand, accept, and engage in a comprehensive discussion regarding their EOL medical decisions. Whenever possible, patients’ primary caregivers should be involved in discussing ADs among patients with terminal illnesses. Future research can explore the EOL care preferences and practices among patients with non-malignant chronic illness and those at different stages of their illness trajectory. A comprehensive understanding of these factors will aid palliative care professionals in promoting AD completion in this population.

### Strength and limitations

Certain limitations of this study exist. First, data were obtained from different hospitals, using diverse data collection methods. Second, this study recorded only ADs’ characteristics and clinical information regarding the last hospital admission before death. Hence, disease-related information, such as the year of disease diagnosis, was not obtained. Third, the characteristics of ADs might not have been completely recorded, if ADs were not signed during the last hospitalization or within the hospital settings. Forth, the majority of ADs were signed by family caregivers in this study, future studies could adopt analysis with only ADs that were signed by patients themselves to allow a more specific analysis. Fifth, it was unlikely to know whether EOL discussions occur after the completion of AD and whether patients’ goals and preferences change over time. Future qualitative studies could explore in this aspect. Lastly, this dataset was collected previously from 2004 to 2009 and might be slightly old, however, it has the potential to demonstrate the influential factors of ADs in Taiwan as this study adopted a nationwide population-based approach.

## Conclusions

Most patients from the reviewed medical records had ADs signed by patients or caregivers, and the most prevalent reasons for signing were patients’ poor prognosis and their imminent death. Patients with non-malignant chronic illnesses were less likely to have signed ADs than those with cancer, with the lowest likelihood observed in patients with cardiovascular diseases. This study highlights the need to explore EOL care preferences and practices among patients with non-malignant chronic illnesses and at different stages of their illness trajectory. Having a higher education level and having others as primary caregivers were also associated with a higher likelihood of signing ADs. Therefore, the involvement of primary caregivers in discussing ADs with patients with terminal illnesses is crucial to AD completion. Furthermore, disclosure of the patients’ poor prognosis should not be overlooked, and there is a need to emphasize the importance of truth telling to the patients’ families and discussing EOL care medical decisions with patients without overriding their wishes. Thus, patients’ preferences toward EOL care medical decisions can be respected, thereby enhancing their quality of EOL care and life before death.

## Data Availability

The datasets used and/or analysed during the current study are available from the corresponding author on reasonable request.

## Abbreviations

AD: Advance directive
CI: Confidence interval
CVI: Content validity index
DNR: Do Not Resuscitate
EOL: End-of-life
OR: Odds ratio

## References

[1] Clements JM (2009). Patient perceptions on the use of advance directives and life prolonging technology. Am J Hosp Palliat Med.

[2] Mukamel DB, Ladd H, Temkin-Greener H (2013). Stability of cardiopulmonary resuscitation and do not resuscitate orders among long-term nursing home residents. Med Care.

[3] Malloy TR, Wigton RS, Meeske J, Tape TG (1992). The influence of treatment descriptions on advance medical directive decisions. J Am Geriatr Soc.

[4] Nicholas LH, Langa KM, Iwashyna TJ, Weir DR (2011). Regional variation in the association between advance directives and end-of-life Medicare expenditures. JAMA.

[5] Teno JM, Gruneir A, Schwartz Z, Nanda A, Wetle T (2007). Association between advance directives and quality of end-of-life care: a national study. J Am Geriatr Soc.

[6] Manu ER, Mody L, McNamara SE, Vitale CA (2017). Advance directives and care received by older nursing home residents. Am J Hosp Palliat Med.

[7] Kim SL, Lee JE, Shimanouchi S (2014). Needs for end-of-life care by home care nurses among non-cancer patients in Korea and Japan. Int J Nurs Pract.

[8] Ostgathe C, Alt-Epping B, Golla H (2011). Non-cancer patients in specialized palliative care in Germany: what are the problems?. Palliat Med.

[9] Fitzsimons D, Mullan D, Wilson J (2007). The challenge of patients’ unmet palliative care needs in the final stages of chronic illness. Palliat Med.

[10] Lau KS, Tse DMW, Chen TWT (2010). Comparing noncancer and cancer deaths in Hong Kong: a retrospective review. J Pain Symptom Manag.

[11] Shin DW, Lee JE, Cho B (2016). End-of-life communication in Korean older adults: with focus on advance care planning and advance directives. Geriatr Gerontol Int.

[12] Keam B, Yun YH, Heo DS (2013). The attitudes of Korean cancer patients, family caregivers, oncologists, and members of the general public toward advance directives. Support Care Cancer.

[13] Rurup ML, Onwuteaka-Philipsen BD, van der Heide A, van der Wal G, Deeg DJ (2006). Frequency and determinants of advance directives concerning end-of-life care in the Netherlands. Soc Sci Med.

[14] Alano GJ, Pekmezaris R, Tai JY (2010). Factors influencing older adults to complete advance directives. Palliat Support Care.

[15] Chu D, Yen Y-F, Hu H-Y (2018). Factors associated with advance directives completion among patients with advance care planning communication in Taipei, Taiwan. Plos One.

[16] Rao JK, Anderson LA, Lin F-C, Laux JP (2014). Completion of advance directives among US consumers. Am J Prev Med.

[17] Van Wijmen MP, Rurup ML, Pasman HRW, Kaspers PJ, Onwuteaka-Philipsen BD (2010). Advance directives in the Netherlands: an empirical contribution to the exploration of a cross-cultural perspective on advance directives. Bioethics.

[18] Kim SH (2011). Factors influencing preferences of Korean people toward advance directives. Nurs Ethics.

[19] Cheng S-Y, Chen C-Y, Chiu T-Y (2016). Advances of hospice palliative care in Taiwan. Korean J Hospice Palliat Care.

[20] Tsai H-H, Tsai Y-F, Liu C-Y (2017). Advance directives and mortality rates among nursing home residents in Taiwan: a retrospective, longitudinal study. Int J Nurs Stud.

[21] Tseng Y-P, Huang L-H, Huang T-H, Hsu L-L, Hsieh S-I (2017). Factors associated with the do-not-resuscitate decision among surrogates of elderly residents at a nursing home in Taiwan. Int J Gerontol.

[22] Andersen RM. Revisiting the behavioral model and access to medical care: does it matter? J Health Soc Behav. 1995:1–0. 7738325

[23] Centers for Disease Control and Prevention (2015). International Classification of Diseases, Ninth Revision (ICD-9).

[24] Polit DF, Beck CT. The content validity index: are you sure you know what’s being reported? Critique and recommendations Res Nurs Health 2006;29(5):489–497. 10.1002/nur.20147 16977646

[25] Fang L, Hsiao L-P, Fang S-H, Chen B-C (2020). Predictors for the intentions of signing advance directives among dialysis patients: a quantitative study. Contemp Nurse.

[26] Lo Y-T, Wang J-J, Liu L-F, Wang C-N (2010). Prevalence and related factors of do-not-resuscitate directives among nursing home residents in Taiwan. J Am Med Dir Assoc.

[27] Mullick A, Martin J, Sallnow L. An introduction to advance care planning in practice. BMJ. 2013;347. 10.1136/bmj.f6064 24144870

[28] Barnes K, Jones L, Tookman A, King M (2007). Acceptability of an advance care planning interview schedule: a focus group study. Palliat Med.

[29] Zhang Q, Xie C, Xie S, Liu Q (2016). The attitudes of Chinese cancer patients and family caregivers toward advance directives. Int J Environ Res Public Health.

[30] Huang C-H, Hu W-Y, Chiu T-Y, Chen C-Y (2008). The practicalities of terminally ill patients signing their own DNR orders—a study in Taiwan. J Med Ethics.

[31] Lovell A, Yates P (2014). Advance care planning in palliative care: a systematic literature review of the contextual factors influencing its uptake 2008–2012. Palliat Med.

[32] Murray SA, Kendall M, Boyd K, Sheikh A (2005). Illness trajectories and palliative care. BMJ.

[33] Butler J, Binney Z, Kalogeropoulos A (2015). Advance directives among hospitalized patients with heart failure. JACC Heart Fail.

[34] Gerald LB, Sanderson B, Fish L, et al. Advance directives in cardiac and pulmonary rehabilitation patients. J Cardiopulm Rehabil Prev. 2000;20(6):340–5. 10.1097/00008483-200011000-00002 11144039

[35] Slavin SD, Warraich HJ (2020). The right time for palliative care in heart failure: a review of critical moments for palliative care intervention. Rev Esp Cardiol (English Edition).

[36] Chang YK, Allen LA, McClung JA, Denvir MA, Philip J, Mori M, Perez-Cruz P, Cheng SY, Collins A, Hui D (2022). Criteria for referral of patients with advanced heart failure for specialized palliative care. J Am Coll Cardiol.

[37] Dunlay SM, Swetz KM, Mueller PS, Roger VL (2012). Advance directives in community patients with heart failure. Circ Cardiovasc Qual Outcomes.

[38] Graphic PC (2015). Dying in America: improving quality and honoring individual preferences near the end of life.

[39] Sulmasy DP, Hughes MT, Thompson RE (2007). How would terminally ill patients have others make decisions for them in the event of decisional incapacity? A longitudinal study. J Am Geriatr Soc.

[40] Rabow MW, Hauser JM, Adams J (2004). Supporting family caregivers at the end of life: they don't know what they don't know. JAMA.

[41] Ryu E-J (2020). Relationships among perceptions of dying well, attitudes toward advance directives, and preferences for advance directives among elderly living alone. Korean J Hospice Palliat Care.

[42] Lynn J, Adamson DM (2003). Living well at the end of life. Adapting health care to serious chronic illness in old age.

[43] Chiu T-Y, Hu W-Y, Cheng S-Y, Chen C-Y (2000). Ethical dilemmas in palliative care: a study in Taiwan. J Med Ethics.

[44] Cheng S-Y, Hu W-Y, Liu W-J, Yao C-A, Chen C-Y, Chiu T-Y (2008). Good death study of elderly patients with terminal cancer in Taiwan. Palliat Med.

[45] Chan CW, Chow MC, Chan S, Sanson-Fisher R, Waller A, Lai TT, Kwan CW (2020). Nurses’ perceptions of and barriers to the optimal end-of-life care in hospitals: a cross-sectional study. J Clin Nurs.

[46] Martina D, Lin CP, Kristanti MS, Bramer WM, Mori M, Korfage IJ, van der Heide A, van der Rijt CC, Rietjens JA (2021). Advance care planning in Asia: a systematic narrative review of healthcare professionals’ knowledge, attitude, and experience. J Am Med Dir Assoc.

[47] Cho CY (2018). From cure to care: the development of hospice care in Taiwan. Palliat Med Int J.

[48] Wang P-Y, Hung Y-N, Smith R (2018). Changes in the use of intensive and supportive procedures for patients with stroke in Taiwan in the last month of life between 2000 and 2010. J Pain Symptom Manag.

[49] Kuo LC, Lee JJ, Cheung DST, et al. End-of-life care in cancer and dementia: a nationwide population-based study of palliative care policy changes. BMJ Support Palliat Care. 2019;12(e3):bmjspcare-2019-001782. 10.1136/bmjspcare-2019-001782 31530554

[50] Kuo L-C, Chen J-H, Lee C-H (2019). End-of-life health care utilization between chronic obstructive pulmonary disease and lung cancer patients. J Pain Symptom Manag.

